# The ethanopharmacological aspect of carbon nanodots in turmeric smoke

**DOI:** 10.1038/srep35586

**Published:** 2016-11-02

**Authors:** Sechul Chun, Manikandan Muthu, Enkhtaivan Gansukh, Pradeep Thalappil, Judy Gopal

**Affiliations:** 1 Department of Bioresource and Food Science, Konkuk University, Seoul 143-701, Korea; 2 DST Unit of Nanoscience (DST UNS), and Thematic Unit of Excellence (TUE), Department of Chemistry, Indian Institute of Technology Madras, Chennai - 600 036, India.

## Abstract

Smoke manifested ever since our ancient’s lit fire; today it has evolved to become an environmental concern. However, medicinal smoke is still part of man’s natural remedies, religious and cultural practices too. The Asiatic household practice of burning turmeric rhizomes to relieve nose and chest congestion is a well known yet never scientifically authenticated or studied practice. For the first time we investigate the components of these turmeric smudges, validate their antimicrobial and anticancer properties and their cell compatibility. With smoke there is always nanoparticulate carbon and turmeric smoke is no exception. If so, what is the role of the nano carbon (NC) in the turmeric smudge effect? This study isolated, characterized and exposed these secret natural NC undercover agents in turmeric smoke. Their unequivocal role in the ethanopharmocological activity of turmeric smudging has been demonstrated. This work opens a new avenue for use of such nano components of smoke for harnessing the ethanopharmacological property of various medicinal smokes, by excluding the smoke factor, through extracting the nano carbon material in them. This is a possibility to realizing the use of such naturally available nanomaterial, as an eco friendly substitute for the notorious anthropogenic nanomaterials.

From time immemorial, smoke has been a part of various areas of human life and culture. Ever since the origin of earth, being generated from natural sources like, hot springs, volcanoes, smoke stealthily grew along with human advancements too. From the first fire lit up by ancient man to all the various things lit up by today’s modern man, smoke has been progressing. Today it has become more of a concern, as a chief pollutant. However, with more knowledge on what smoke is, man went on to exploit the goodness of smoke, as in case of burning of various herbs and wood for natural remedies and in rituals. This is what came to be known as ‘medicinal smoke’; known for its varied healing properties. Thus, medicinal smoke percolated not just human welfare and health, but also human culture and religion too.

In early Indian writing (Sushruta 800–600 BC), the fumigation of an operating room with fumes of mustard, butter and salt was considered an early form of antisepsis” of the air, although it was primarily used to get rid of evil spirits. During 522–486 BC, smoke by burning esfand (*Peganum harmala*) and/or sandalwood (*Santalum album*) was used to protect the king from evil and disease. Throughout the medieval period, including the terrible years of the bubonic plague caused by the causal bacterium *Yersinia pestis*, the main prophylactic measure against infectious diseases was fumigation by burning incense, herbs and aromatic essences. The burning of herbs and plant parts for medicinal and spiritual purposes which is the so-called ‘smudging’ – is an ancient practice among indigenous people around the world, while the cultural and religious use predominates Asian countries. More than 50 countries across 5 continents are therefore using medicinal smoke with some motive or the other.

Researchers have found that, medicinal smoke is mostly used to address the following specific organ systems: “pulmonary (23.5%), neurological (21.8%) and dermatological (8.1%)”. They also found that “ambient smoke”, which is the type of passively inhaled smoke generated by smudging/incense, is believed to be an effective “air purifier”^1^. It is even expected that modern medicine would delve into the usage of medicinal smoke as a drug delivery system, owing to reasons such as: rapid delivery to the brain, more efficient absorption by the body and lower costs of production. It is well known that, smoke produced at high temperature was found to be one of the simple ways of drug administration. In some cultures, the use of smoke is not limited to medical applications but is part of religious celebrations and ceremony. Smoke produced from natural substances has been used extensively in many religious rituals. Famous ancient physicians have described and recommended this practice. Mohagheghzadeh *et al*.^2^ have summarized an exhaustive review on medicinal smoke, their source and applications. In their review they list out that species from only four families make up a little over one-third (35.9%) of the list: Asteraceae (10.6%), Solanaceae (10.2%), Fabaceae (9.8%) and Apiaceae (5.3%). Their Leaves (29.1%), fruit (15.3%), whole plants (12.5%), underground organs (12.1%) were identified as the major sources of medicinal smoke. These plants yielding medicinal smoke have diverse applications: as air purifier, dermatological conditioners, febrifuges and disinfectants, for gastrointestinal conditions, genito-urinary conditions, mood disorders, neurological conditions, orthopaedic conditions, pulmonary conditions, toothache and other problems of the mouth, treatment of the ear, treatment of the eye and other purposes^1^.

Chemically, smoke is a gaseous product of incomplete combustion of organic substances, such as wood, coal and tobacco, and is chiefly made up of suspended particles of unburnt carbon, which settle as soot (The Encyclopedia Americana, 1963). Thus, with the major composition in smoke being carbon, there is a high probability that this carbon is present in nanoform^3^ and also that this carbon nanomaterial may be functionalized with the active components contained in these medicinal smokes and thus play a role in the medicinal property exhibited by the medicinal smoke. This is the speculation that motivated this investigation.

Turmeric is a spice that is commonly used in Indian curries. It grows in India, China and other Asian countries, and has been used for centuries in the natural healing system from India, called Ayurveda. Turmeric is a powerful anti-inflammatory, antiseptic and disinfectant and is used in several different ways, including burning and inhaling the smoke. The burning of turmeric rhizomes and inhaling the smoke is a very common practice in India. A practice which has been handed down traditionally from grandmothers, mothers to daughters, for immediate relief from chest and nose congestion in infants. Some of the acquired knowledge includes; (a) inhaling smoke from burning turmeric to relieve congestion due to chronic colds or cough, breathing in through each nostril individually for the smoke to release large amounts of mucous from the sinus cavity, (b) burning dry turmeric rhizome dipped into coconut or neem oil for relief from sinusitis and (c) inhaling turmeric smoke to bring an end to hiccups. A “hysterical fit”, or fit of hysteria typically involves strong, emotionally irrational behavior and outbursts that may be violent. Inhaling the smoke of a burning turmeric plant will put an end to hysterical fits. In all of these usages, the relief factor is almost in seconds, spontaneous. While reviewing the literature, it was surprising to see that the records for burning of turmeric for medicinal properties are more of blogs and private writings, seconded by almost no scientific publication. This actually is the motivation for this study, to talk about the science behind this traditional practice, to nail the components that are involved and to expose the nano contribution aspect.

In the current report, we are out to prove the nano carbon influence on the medicinal property of the smoke in medicinal smoke. For the first time, we would attempt isolation, characterization, and application of the carbon nanomaterial from turmeric smudges. We would also prove the antibacterial effect of turmeric smudges and validate the crucial role that the nano carbon play in the ethanopharmacological aspect of turmeric smudges.

## Materials and Methods

### Chemicals

Turmeric rhizomes were purchased from a local supermarket in Seoul, Korea. All the chemicals used in the study, unless specified as otherwise, were all of analytical grade. Millipore water was used for all experiments.

### Sample Preparation

**Figure 1:**
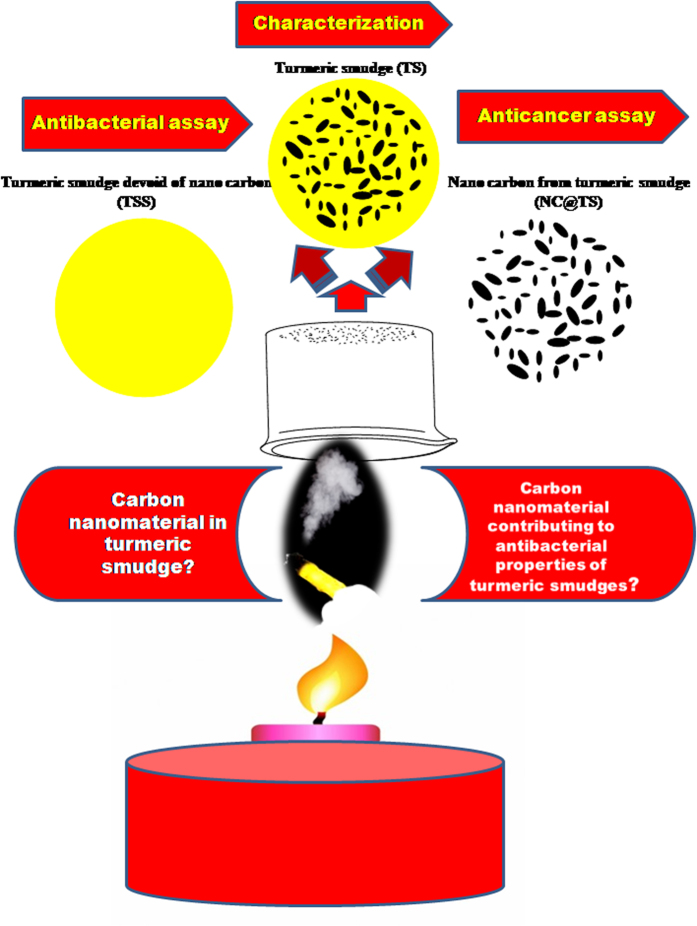
Scheme showing the workflow of the study.

The turmeric rhizomes were burnt to red hot using a spirit lamp in a laminar flow and the fumes collected using an inverted sterile glass beaker. Five rhizomes about the size of 5 cm each were used per batch of sample preparation. The soot settled on the sides of the beaker was detached ultrasonically (JAC-2010, KODO Technical Research Co., Ltd, Hwaseong-City, Gyeonggi-Do, Korea) into 10 mL of sterile water, by sonication for 10 min. The carbon soot samples were stored under refrigeration upto further use. In order to separate out the carbon nanoparticles from the carbon soot suspension, the suspensions were centrifuged (Beckman Coulter Avanti J-25, USA) at 19,000 rpm for 1 h. After centrifugation the supernatant and the settled black precipitated carbon particles were separately collected and stored. Figure 1 gives the schematic representation of the sample preparation methodology. Three different test conditions were used for the testings reported below: TSS (turmeric smudge supernatant, without nano carbon material), is the turmeric smudge suspension rid of the nano carbon material by centrifugation as mentioned above, TS (turmeric smudge without centrifugation) which is the turmeric smudge with the nano carbon material and NC@TS, which is the carbon material harvested by centrifugation from the turmeric smudge. These codes will be used to discuss the results in the following sections.

### Characterization

The turmeric smudge (TS) was characterized for the presence of carbon nanomaterial using a Nanodrop ND-1000 v 3.3.1 spectrophotometer, (Nanodrop Technologies, Inc., Wilmington, USA). The absorbance was scanned from 220–700 nm. The NC@TS was characterized using a JEM-1400PLUS, transmission electron microscope (TEM), JEOL USA, Inc. Peabody, MA, USA. Further characterization was done using FTIR (Shimadzu FTIR-8300 spectrometer, San Diego, CA, USA) using KBr pellets. For FTIR, KBr was added directly into the beaker containing the smudge collected from the turmeric fumes, and then the smudge was scraped out with mixing alongwith the KBr powder. When the KBr powder took up the turmeric smudge, it turned grayish. After this, it was ground and pellets were made as per routine FTIR analysis. FTIR of the carbon particles extracted via centrifugation (NC@TS) was conducted too. In this case the samples were dried in an oven and the powder was used for analysis. Energy Dispersive X-ray analysis (EDAX) was used to confirm the chemical composition of the TS using an FE-SEM ((JEOL, JSM-5410LV).

### Antibacterial Testing

The antibacterial activity of all three test variants, TSS, TS and NC@TS was determined via turbidometric assay using the spectrophotometer and also total viable count method using plate count technique. Five bacterial strains: *Streptococcus mutans*, *Salmonella enteritidis*, *Staphylococcus aureus*, *Pseudomonas aeruginosa* and *Escherichia coli* were used for the antibacterial tests. *Escherichia coli* (ATCC 11234), *Salmonella enteritidis* 12021 (IFO 3313) and *Streptococcus mutans* 11823 (ATCC 25175) were purchased from Korean culture centre of microorganisms, Seoul, South Korea. *Staphylococcus aureus sub*. *aureus* (KCTC 1928) and *Pseudomonas aeruginosa* (KACC 11085) were obtained from Korean collection for type cultures, Seoul, South Korea. *S*. *mutans* was cultured on Bacto^TM^ Brain Heart Infusion broth and BBL^TM^ Brain Heart Infusion Agar and *P*. *aeruginosa* was cultured on Difco^TM^ Nurtient Broth and Difco^TM^ Nutrient Agar and *Salmonella enteritidis*, *Staphylococcus aureus* and *Escherichia coli* were cultured on Difco^TM^ Luria-Bertani broth and Difco^TM^ Luria-Bertani Agar.

10 mL quantities of the appropriate culture medium was inoculated with the respective bacterial strain and the respective test variant, such as TSS, TS or NC@TS (at concentrations of 100 μL/mL) added and incubated in a shaker cum incubator at 37 °C overnight. Absorbance was measured at 600 nm using a spectrophotometer. The total viable count (TVC), indicating the number of bacteria that survived after the interaction, were enumerated my plate count method. The TVC was represented as cfu/mL (colony forming unit/mL)^4^,^5^.

### Cell compatibility Testing

In order to test the cell compatibility of TSS, TS and NC@TS, their cell interaction properties were assessed. The cytotoxicity assay/cell compatibility assay was conducted as previously described by Enkhtaivan *et al*.^6^ using Madin-Darby Canine Kidney (MDCK) cell lines (ATCC, CCL-34) purchased from American Type Tissue Culture collection, USA. 1.5 × 10^4^ cells per mL were seeded onto 96 well plates and maintained in 37 °C, 5% of CO_2_ in a humidified incubator. After 24 hours of incubation, the media was removed and washed twice with PBS solution. Eight different concentrations (30 μL/mL, 40 μL/mL, 50 μL/mL, 60 μL/mL, 70 μL/mL, 80 μL/mL, 90 μL/mL and 100 μL/mL) of the respective TSS, TS and NC@TS test variants in triplicates were added onto individual wells and replenished with fresh media. After incubating for 48 hours, the medium was removed and washed twice with PBS. Washed plates were fixed with 70% of acetone for 1 hour in -4 °C. Then the acetone was removed and plates were dried in a drying oven at 60 °C. After drying, SRB assay was performed for determining cell toxicity. 100 μL/mL of SRB solution (0.4% w/v) was added into each well and incubated overnight at room temperature. After the SRB solution was discarded, plates were washed 5 times with 1% of acetic acid and then dried using a drying oven. SRB stained 96 well plates were incubated overnight in 10 mM of tris-base. After the SRB had completely dissolved in tris-base, the absorbance was read at 540 nm and the percent inhibition was calculated. One set of plates were retained after incubation with the test substrates for microscopic imaging.

### Anticancer activity

In case of anticancer activity, a similar protocol was followed as in case of the cell compatibility assay, the only difference being the use of Caki 2 cell line (Caki-2 ATCC^®^ HTB-47™) purchased from American Type Tissue Culture collection, USA.

### Fluorescence Imaging

A fluorescence microscope (Axiovert 2000, Carl Zeiss, Germany) was used to determine the ability of the nano carbon in the smudges to assist in fluorescence imaging of the cells. After overnight incubation with NC@TS, the cells were imaged under a fluorescence microscope.

## Results

### Characterization of the Turmeric smudges

**Figure 2:**
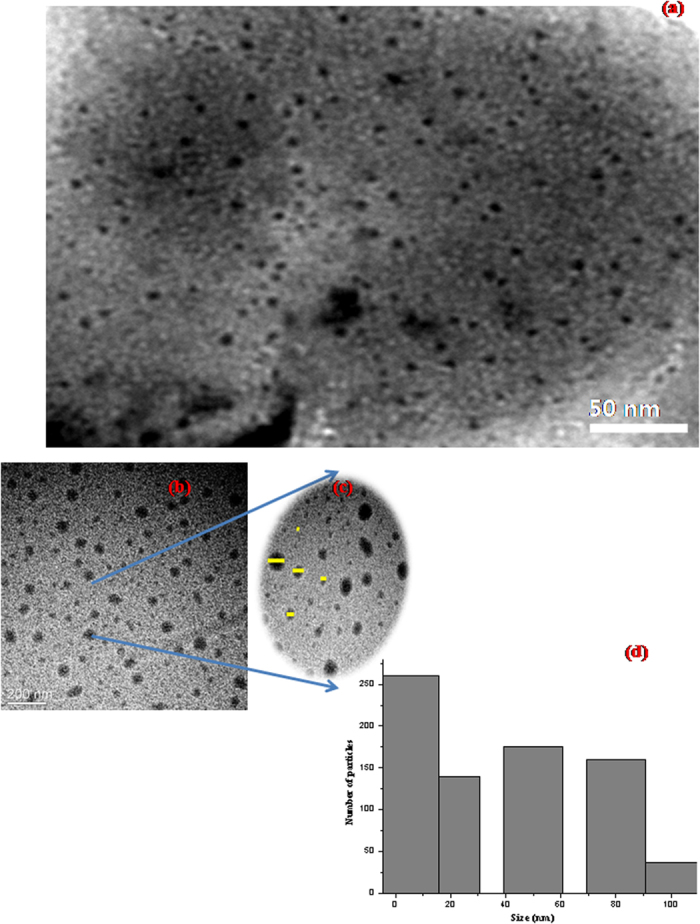
TEM image showing (**a**) predominating 5–10 nm sized particles (**b**) 20–25 nm, 50 nm, 80 nm and 100–300 nm particles, (**c**) the five different sized particles highlighted (**d**) Particle size distribution histogram.

The turmeric smudges (TS) were characterized using HR-TEM. HR-TEM images revealed the presence of abundant spherical to oval shaped nanoforms in the turmeric smudges. The sizes of the nanoforms varied from 5 nm to 500 nm. Figure 2(a) shows 5–10 nm sizes predominating in the TS. Figure 2(b) displays the overall view. As can be observed from Fig. 2(c) five size ranges of the particles were evident. Particle sizes were predominantly in the size range of 5–10 nm, 20–25 nm, 50 nm, 80 nm and 100–300 nm. The size regime from 5–10 nm predominated, as depicted by the particle size distribution histogram in Fig. 2(d). This irregular particle size distribution is a common observation in terms of naturally occurring nanoparticles. Also, more so in nanomaterial present in soot, as reported by Anu and Manoj^7^, Song *et al*.^8^, and David *et al*.^9^.

**Figure 3:**
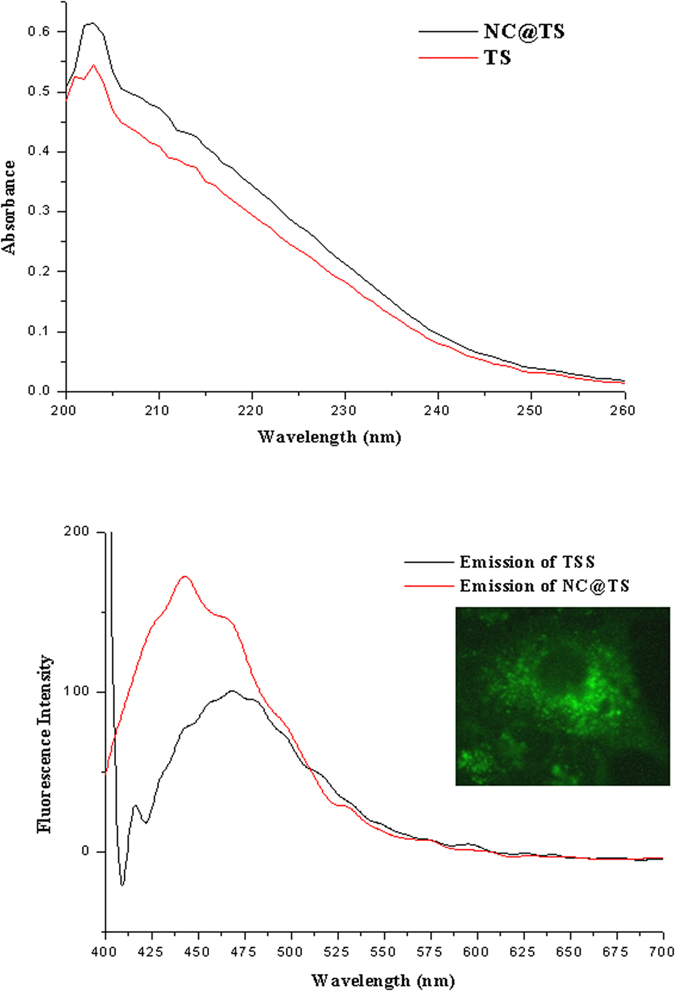
(**a**) UV-Vis spectra and (**b**) Fluorescence spectra of the turmeric smudge test components (inset showing fluorescence microscopic image of the NC@TS particles at 420–470 nm).

The absorption property of the TS was characterized using UV-Vis spectroscopy. An absorption maximum is clearly observable in the UV range of the spectrum between 206 nm and 251 nm from the TS sample (Fig. 3a). The UV-Vis properties of NC@TS precipitated out of the turmeric smudges was also studied. It was interesting to observe that the absorption properties of the precipitated nano carbon (NC@TS) and that of TS were similar and matched the absorption characteristics of carbon nanoparticles obtained from natural gas soot^8^. It was interesting to observe that these soot derived carbon nanoparticles showed highly identical absorption characteristics.

The fluorescing properties of NC@TS were also studied and were reported to show emission at 450 nm (Fig. 3b) which is similar to that of fluorescent carbon nanomaterial reported in previous studies^10^. The smaller shoulder was also observed at 475 nm, which is possibly from the curcumin fractions of the turmeric, which also possess fluorescent properties. In TSS (which is the supernatant after removal of the nano carbon), the 450 nm peak was absent and the 475 nm peak was more pronounced. This additionally confirms the 450 nm emission to be from the NC@TS. The inset in Fig. 3b shows the fluorescence exhibited by the NC@TS at 420–470 nm as captured by a fluorescence microscope.

**Figure 4:**
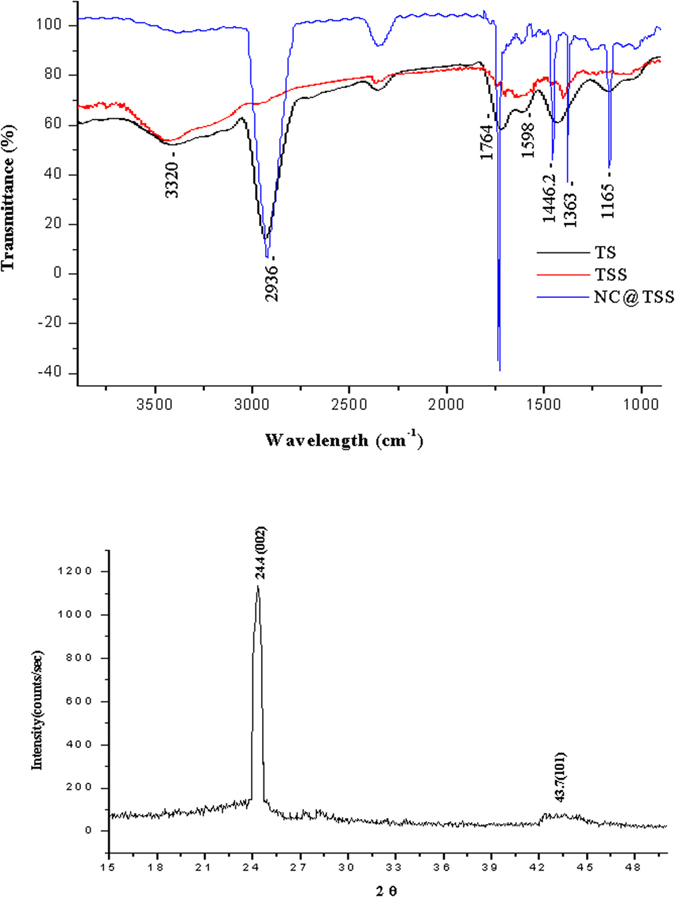
(**a**) FTIR spectra of the turmeric smudge test components. (**b**) XRD of NC@TS confirming the presence of graphitic carbon.

FESEM based EDAX was also conducted to analyze the elemental composition of TS. Table 1 presents the results of the EDAX analysis. As observed from the table, the major element was carbon (68%) in the turmeric smudges. This further confirms the chemical composition of the turmeric smudge and the presence of significant amount of carbon in them. FTIR was conducted to confirm the chemical nature and functionalization of the turmeric smudge derived nanomaterial (Fig. 4a). Previous researchers^8^ have indicated that characteristic absorption bands of υ (O-H), υ (C-H) and υ (C = O) at 3320 cm^−1^, 2934 cm^−1^ and 1764 cm^−1^, respectively, conform to the existence of –COOH in case of carbon nanoparticles. The peak position at around 2360 cm^−1^ is due to the presence of CO_2_ and 1165 cm^−1^ the C = O (Stretch). Our FTIR results (Fig. 4a) show these above mentioned absorption bands identical to that expected of carbon nanoparticles in TS and NC@TS. As observed in Fig. 4a, the removal of carbon nanoparticles resulted in a sharp decrease in the intensity of the absorption bands in TSS. Further, it was also observed that the NC@TS were also functionalized with reputed antimicrobial essential oil components that exist in turmeric. The FTIR also showed absorption bands matching that of tumerone, which is reported to constitute about 60% of the essential oil in turmeric. Using Ominic 7.4 (Thermo Fischer Scientific Inc., Nicolet 6700, Firmware version 2.09) we correlated our spectra with the online library, the results revealed that Dimethylcyclopenanone, Butylcyclohexanone, trimethylhexanal and their equivalents were functionalized on the turmeric smudge derived carbon dots. It is crucial to observe from Fig. 4a that the FTIR spectra of the TS (which is the smudge collected from the burning of the rhizome) and the NC@TS (the separated nano Carbon) showed similar spectral pattern with identical absorption bands. As observed from Fig. 4a, the NC@TS showed significantly high intensity bands matching that of turmeric essential oil components/sesquiterpenes including tumerone derivatives (heptane,2,2,4,6,6-pentamethyl-, 3-heptene,2,2,4,6,6-pentamethyl-, 2,2,4,6,6-pentamethylheptane) and other sesquiterpenoid ketones/lactones such as dimethylcyclopenanone, tetramethylcyclohexanone, butylcyclohexanone, dimethylnonanone and trimethylhexanal. This additionally confirms that these bioactive components in the smudges were functionalized onto the NCs. This is probably why the NCs exhibited properties similar to that of turmeric smudges. Table 2 summarizes the list of compounds matched through the FTIR library, presenting the match percentage.

Figure 4b gives the results of the XRD characterization of the NC@TS, as observed from the figure, a high-intensity diffraction peak at 2θ = 24.4 and one additional peak at 2θ = 43.7 that are ascribed to (002) and (101) diffraction of graphitic carbon respectively were obtained^3^. These results once again validate the presence of carbon in NC@TS.

### Antibacterial Properties of TSS and NC’s

**Figure 5:**
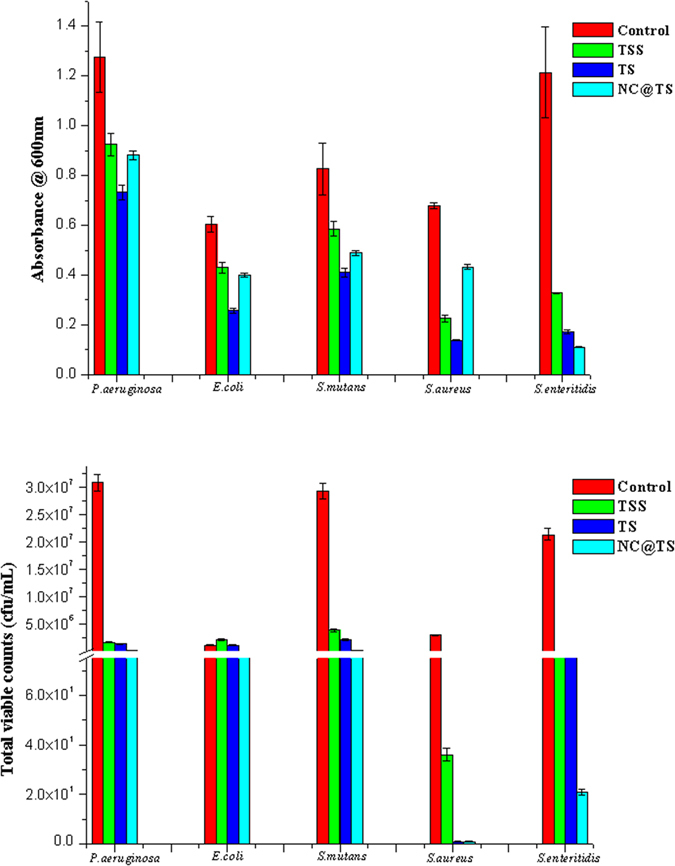
Graph showing results of antibacterial assay (**a**) spectrophotometric method (**b**) plate count method.

The antibacterial efficiency of the three turmeric smudge test components (TSS, TS and NC@TS) was evaluated against five predominant bacteria, *S*. *mutans*, *S*. *enteritidis*, *S*. *aureus*, *P*. *aeruginosa* and *E*. *coli*. Figure 5a,b presents the results of this study. As observed from these figures, both spectrophotometric turbidity method (Fig. 5a) and total viable count method (Fig. 5b), demonstrated that the turmeric smudges possessed significant antibacterial activity. The results demonstrated that within the test bacteria, *S*. *aureus*, was the most susceptible, followed by *S*. *mutans*, *S*. *enteritidis*, *and P*. *aeruginosa*, however, the least susceptible was *E*. *coli*. A distinct decrease in antibacterial activity was observed with respect to TSS, which was rid of the nanocarbon components. Compared to TS it was observed that the isolated NC@TS showed significant antibacterial activity. These results go on to further confirm the significant contribution of the NCs in the turmeric smudge towards its antibacterial activity.

### Cell compatibility

**Figure 6:**
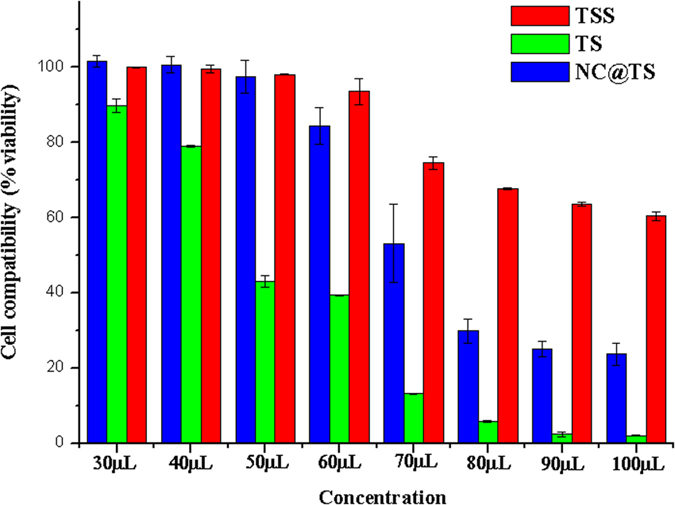
Cell compatibility results of TS, TSS and NC@TS against MDCK cells.

**Figure 7:**
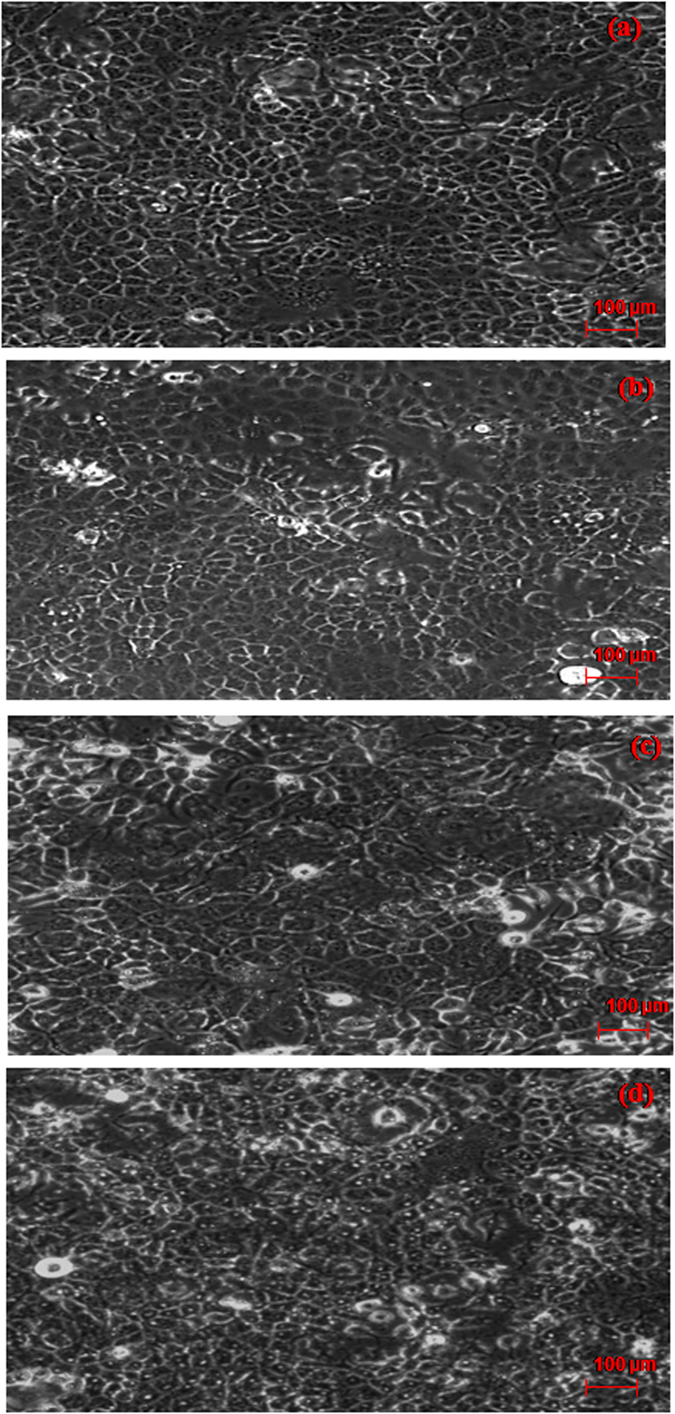
Transmission image of the cells following treatment with NC@TS.

Cell compatibility of TSS, TS, NC@TS was studied in order to assess their effect on normal human cells (Fig. 6). Since, it is a mandatory prerequisite that any anticancer agent that is used in the human system should not harm the normal healthy cells. The cell compatibility here is represented by the test variables not affecting the normal cells, as shown by the cell viability remaining unaffected. A decrease in the cell viability denotes that the cell compatibility has been compromised. It was observed that TSS was compatible even upto concentrations as high as 100 μL/mL. In case of the NC@TS they also exhibited significant compatibility (upto 70 μL/mL) with human cells. But with respect to TS, which is the actual traditionally used medicinal smoke system, poor cell compatibility was observed (40 μL). Even concentrations as low 40 μL/mL were found to affect the normal cells, as shown by the abrupt decrease in cell viability in Fig. 6. Figure 7 is the optical micrograph of the cells after exposure to NC@TS at 40 μL/mL (Fig. 7(a)), 50 μL/mL (Fig. 7(b)), 60 μL/mL (Fig. 7(c)) and 70 μL/mL (Fig. 7(d)) concentrations, showing good cell adherence. Dead cells if present would be evident as cells that have lost their adherence and therefore are afloat the culture media in the plates. As observed from the figure at all these concentrations NC@TS showed excellent biocompatibility.

### Anti cancer activity

**Figure 8:**
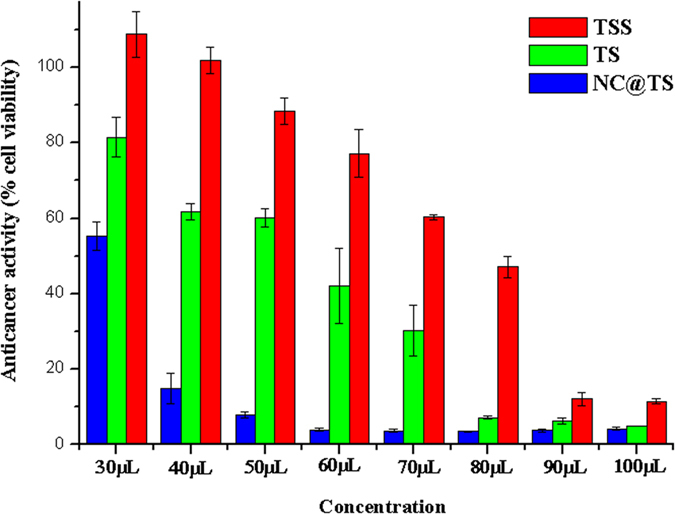
Graph showing results of anticancer assay against Caki 2 cell lines.

**Figure 9:**
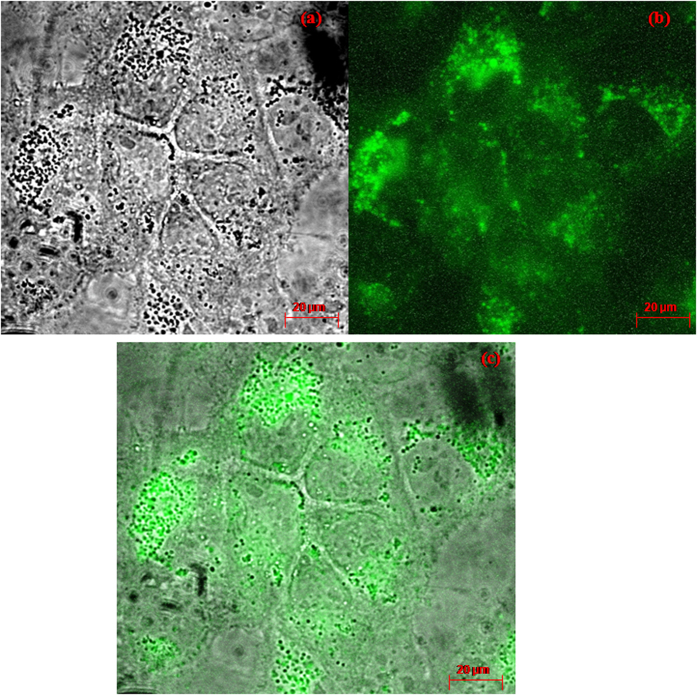
Fluorescence images of the nano carbon interacted MDCK cells (**a**) brightfield (**b**) fluorescence at 420–470 nm, (**c**) superimposed.

The anticancer activity of the test components was also tested (Fig. 8). The decrease in the viability of the cancer cells is proportional to the anticancer ability of the test component. The NC@TS showed significantly high anticancer activity at concentrations as low as 40 μL/mL, while the TS showed significant anticancer activity only beyond 60 μL/mL concentrations. At all concentrations TSS showed merely marginal activity. In addition, it was observed that the NC@TS were found to easily travel across cell barriers, Fig. 9(a–c) shows the localization of the NCs within MDCK cells. Also, the possible use of these NCs in fluorescent imaging is demonstrated in Fig. 9(b,c). The particles were operational at 420–470 nm.

## Discussion

Burning of turmeric rhizomes to ward away nose congestion and common cold related discomfort of the nasal passage is a common practice within households. Apart from this, turmeric rhizomes are used in various ceremonies and rituals in various cultures across Asia. The motivation for this work comes as an outcome of the understanding that carbon nanomaterials are a major component of smoke soot^8^,^10^ and if so, what is their role in turmeric smudges. In addition to that, it was rather unusual to observe that antibacterial activity of turmeric smudges has till date remained a household phenomenon and has not been authenticated by scientific investigations. Our quest to find answers, led us to this point where based on the results: (a) carbon nanomaterial were obtained in abundance from the turmeric smudge, (b) these nano carbon entities (NC@TS) were functionalized with antimicrobial essential oils, (c) these NCs played a very significant role in the antibacterial activity and (f) the antibacterial activity reduced with the removal of NCs from the smudges.

### Modulus Operandi of NC@TS

Although we have not provided direct experimental results towards the mechanism of antibacterial/anticancer activity of the NCs, based on FTIR and fluorescence microscopic evidences we speculate the probable mechanism behind the activity. The antibacterial activity of turmeric is owing to the presence of Curcumin or diferuloylmethane (1,7-bis(4-hydroxy-3-methoxyphenyl)-1,6-heptadiene-3,5-dione) and curcuminoids and essential oil components. In, the current study although curcumin is not expected to show up, owing to the high temperature incineration process, therefore it is the volatile essential oils that are playing the crucial role in the bactericidal activity. The major compounds identified in turmeric volatile oil are turmerone, turmerone, curcumene, zingiberene, α-phellandrene, curlone, 1,8-cineol and some other sesquiterpenes. Turmerones are sesquiterpenoid cyclic ketones that account for 40–50% of the volatile oil^11^ (Govindarajan and Stahl). Turmerones are the major constituents present in oil and possess many biological activities including antibacterial activity^12^,^13^,^14^,^15^,^16^,^17^. As observed from Table 2, the FTIR results revealed that all the components present in the turmeric smudge (TS) were obtained functionalized on the isolated nano carbon surfaces too.

In general, smoke arises from burning contains carbon particles, when we obstruct the path of the rising smoke with a substrate these will condense on the surface forming black soot. In our case when we burn the rhizome the smoke that arises consists of other volatile oils too and when they deposit they deposit together with the carbon in the soot. Then when the soot is eluted into water, these hydrophobic volatile oil components find a better harbor on the carbon particles than in water, leading to the functionalization that we observe in this case. With the added advantage of the increased surface area in case of nanosized carbon in the soot, enhanced sites were available for functionalization of the volatile oils. The size, the enhanced absorption across cell membranes, the functionalization of bioactive components, all combine to aid in the success of the NC@TS in killing the bacteria.

**Figure 10:**
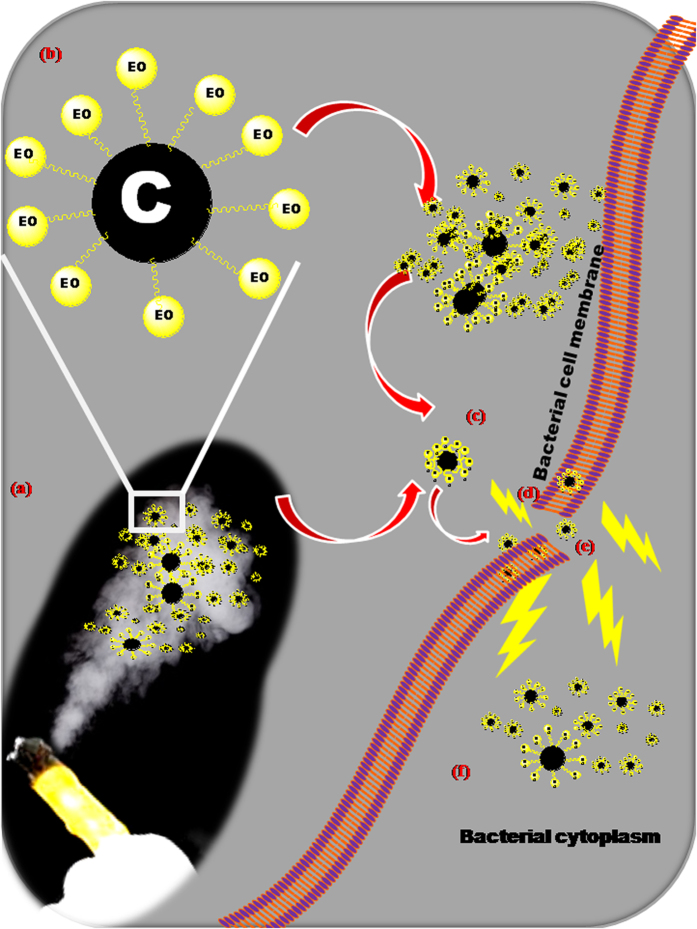
Speculated modulus operandi of the nano Carbon (NC) in the turmeric smoke against bacterial cells.

In an excellent review, Burt^18^ has reviewed the reports on the mode of action of various essential oils^18^. Based on the review, EO components’ site of action in bacteria include: degradation of the cell wall^19^,^20^; damage to cytoplasmic membrane^21^,^22^,^23^,^24^; damage to membrane proteins^25^,^26^; leakage of cell contents^20^,^23^,^27^,^28^,^29^; coagulation of cytoplasm^27^ and depletion of the proton motive force^30^. The capping of essential oils (EO) on nanocarbon particles was an even added advantage, enabling the easy passage of the EO into the bacterial cell. Generally, essential oils are reported^31^ to exhibit stronger antimicrobial activity on Gram-positive bacteria (compared to Gram-negative bacteria) because of the presence of lipoteichoic acids, in the (less complex, single-layer) membranes that facilitate the penetration of hydrophobic compounds present in essential oils. These compounds first site of attack is the membrane, where they affect the membrane permeability, consequently inducing leakage of ions and important cell contents, finally leading to the cell death. On the other side, it is reported that the Gram-negative bacteria are resistant due to the presence of extrinsic membrane proteins or cell wall lipopolysaccharides, which are able to limit the diffusion rate of hydrophobic compounds through the (more complex, double layered lipopolysaccharide) cell membrane^18^,^32^. However, in our case, since the EO were capped onto the nano carbon in the turmeric smudges, their passage across the cell membrane was enabled owing to the nano size dimensions of the carbon, effecting antibacterial activity on gram negative bacterial species too. Figure 10 presents the speculated mode of action and the sequence of modulus operandi by the NC@TS. As shown in the figure, the primary site of attack is the cell membrane (Fig. 10(d)), which is also the first defense of the bacterial cell. Affecting the cell membrane affects the integrity and functionality of the membrane (Fig. 10(e)). The fluorescence microscopic imaging of the cells (Fig. 9(a)) also indicates the NCs were localized more at the cell membrane and lesser inside the cells. The NCs also have better access than EO across the membranes owing to their nano dimensions, once inside (Fig. 10(f)); they trigger off a cascade of other attacks on the cytoplasm and organelles, resulting in cell death. It is also interesting to note here that Sheena *et al*.^33^, have reported that kitchen soot derived carbon nanoparticles possessed inherent antibacterial property. Thus, it appears that a combination of carbon nanoparticle property and the EO functionalization with the added advantage of enhanced absorption across the cell membrane is key to the observed activity.

In addition, as demonstrated by the cell compatibility tests, the turmeric smudges do possess considerable amount of cell toxicity too, which is possibly due to the smoke effect. With the known popularity of carbon for its biocompatibility^3^,^33^, which was also tested and confirmed in case of the turmeric smudge derived NC@TS obtained in our study too, it definitely holds an edge to turmeric smoke. This study confirms that harvesting the NC@TS could lead to safer and effective extension of this age old smudging method to real time drug delivery and pharmaceutical applications.

## Conclusion

The results of this study successfully isolated nano carbon from turmeric smudges and characterized the obtained NC@TS. The antibacterial, anticancer ability of the TS was compared against NC@TS. The superior cell compatibility of NC@TS was proved against the conventional TS widely used in smudging. For the first time we expose these naturally obtained NCs which hold a huge potential and bioactive properties to a promising future in the realms of drug delivery and biomedical applications.

## Additional Information

**How to cite this article**: Gopal, J. *et al*. The ethanopharmacological aspect of carbon nanodots in turmeric smoke. *Sci. Rep.* **6**, 35586; doi: 10.1038/srep35586 (2016).

**Publisher’s note:** Springer Nature remains neutral with regard to jurisdictional claims in published maps and institutional affiliations.

## Figures and Tables

**Table 1:** FE-SEM EDAX results of turmeric smudge.

Element	Series	Norm. C (Wt%)	Atomic. C (Wt%)
C	K	68.19	78.03
O	K	23.02	19.77
Al	K	1.46	0.75
Na	K	0.76	0.45
Pt	M	4.95	0.35
Si	K	0.73	0.28
Cl	K	0.43	0.21
K	K	0.46	0.16
	Total	100	100

**Table 2:** Results of the OMNIC 7.4 library search of TS and NCs@TS.

Sample Source	Library Index	Match (%)	Compound Name	Library Name
TS, TSS, NC@TS	1843	70–79	3,3,3,5-Tetramethylcyclohexanone	Nicolet/AldrichVapor phase
TS, NC@TS	1771	75–76	4-t-butylcyclohexanone	Nicolet/AldrichVapor phase
TS, NC@TS	605	67–74	3,5,5-trimethylhexanal	Flavors and fragrances
TS	2638	73	2,5-dimethylcyclopentanone	
TS, NC@TS	3056	67–68	2,8-dimethyl-5-nonanone	Nicolet/AldrichVapor phase
TS, NC@TS	737	67–68	5-nonanone,2,8-dimethyl	EPA Vapor phase
TS, NC@TS	3028	67.25	Isopentyl disulfide	EPA Vapor phase
TS, NC@TS	1874	69	4-t-amylcyclohexanone	Nicolet/AldrichVapor phase
TS, NC	554	68	4-Heptanol,2,6-dimethyl-	EPA Vapor phase
TS, NC	2917, 994, 993	69	heptane,2,2,4,6,6-Pentamethyl-, 3-heptene,2,2,4,6,6-pentamethyl-, 2,2,4,6,6-pentamethylheptane	EPA Vapor phase
